# Performance of machine translators in translating French medical research abstracts to English: A comparative study of DeepL, Google Translate, and CUBBITT

**DOI:** 10.1371/journal.pone.0297183

**Published:** 2024-02-01

**Authors:** Paul Sebo, Sylvain de Lucia

**Affiliations:** 1 University Institute for Primary Care (IuMFE), University of Geneva, Geneva, Switzerland; 2 Geneva University Hospitals, Geneva, Switzerland; COMSATS University Islamabad, PAKISTAN

## Abstract

**Background:**

Non-English speaking researchers may find it difficult to write articles in English and may be tempted to use machine translators (MTs) to facilitate their task. We compared the performance of *DeepL*, *Google Translate*, and *CUBBITT* for the translation of abstracts from French to English.

**Methods:**

We selected ten abstracts published in 2021 in two high-impact bilingual medical journals (*CMAJ* and *Canadian Family Physician*) and used nine metrics of *Recall-Oriented Understudy for Gisting Evaluation* (*ROUGE-1 recall/precision/F1-score*, *ROUGE-2 recall/precision/F1-score*, and *ROUGE-L recall/precision/F1-score*) to evaluate the accuracy of the translation (scores ranging from zero to one [= maximum]). We also used the *fluency score* assigned by ten raters to evaluate the stylistic quality of the translation (ranging from ten [= incomprehensible] to fifty [= flawless English]). We used Kruskal-Wallis tests to compare the medians between the three MTs. For the human evaluation, we also examined the original English text.

**Results:**

Differences in medians were not statistically significant for the nine metrics of *ROUGE* (medians: min-max = 0.5246–0.7392 for *DeepL*, 0.4634–0.7200 for *Google Translate*, 0.4815–0.7316 for *CUBBITT*, all p-values > 0.10). For the human evaluation, *CUBBITT* tended to score higher than *DeepL*, *Google Translate*, and the original English text (median = 43 for *CUBBITT*, vs. 39, 38, and 40, respectively, p-value = 0.003).

**Conclusion:**

The three MTs performed similarly when tested with *ROUGE*, but *CUBBITT* was slightly better than the other two using human evaluation. Although we only included abstracts and did not evaluate the time required for post-editing, we believe that French-speaking researchers could use *DeepL*, *Google Translate*, or *CUBBITT* when writing articles in English.

## Introduction

The dominance of English as the publishing language can penalize non-English speaking researchers seeking to share their work, as the stylistic quality of articles can have an impact on their likelihood of being published and/or cited by other publications [1]. In order to improve their chances of publication in English-language journals, researchers often rely on professional translation services to improve the style of their articles before submission [1]. However, these services are expensive, use translators who are not necessarily experts in the field, and are time-consuming, which often greatly delays the submission of articles [1].

Machine translators (MTs) are increasingly used in everyday life [2, 3]. Indeed, thanks to neural networks, the quality of translation has greatly improved in the last decades [4–6] and they do not require advanced computer skills. They are also used in medicine, for example to translate electronic medical records and to improve patient management in clinical practice, with mixed results [3, 7–15]. For example, Taira et al assessed the use of *Google Translate* for translating commonly used Emergency Department discharge instructions into seven languages [9]. While the overall meaning was retained in 82.5% of translations, accuracy rates varied across languages, and the study concluded that *Google Translate* should not be relied upon for patient instructions due to inconsistency in translation quality. In another study, Turner et al assessed the feasibility of using *Google Translate* followed by human post-editing to translate public health materials from English to Chinese [10]. The results showed that common machine translation errors and challenges in post-editing led to lower quality translations, suggesting the need for improvements in machine translation and post-editing processes before routine use in public health practice. However, a previous study of the same research team suggested that *Google Translate* and post-editing could yield translations of comparable quality in a more efficient and cost-effective manner for English to Spanish [11]. Blind ratings by two bilingual public health professionals indicated that when comparing human translation and machine translation followed by human post-editing, both types of translations were considered overall equivalent, with 33% preferring human translation, 33% preferring machine translation followed by human post-editing, and 33% finding both translations to be of equal quality. According to the authors, these divergent results between the two studies are linked to significant differences between English and Chinese, for example in syntactic structures. Khoong et al also found marked differences between Spanish and Chinese when using *Google Translate* for translations of emergency department discharge instructions [12]. Among the 100 sets of patient instructions containing 647 sentences, *Google Translate* accurately translated 92% of sentences into Spanish and 81% into Chinese. A minority of the inaccuracies in the translations had the potential for clinically significant harm.

Only a few studies evaluated the use of MTs in academic research, and they mainly focused on the extraction of relevant data from non-English articles [16–18]. For example, Balk et al compared *Google Translate*’s ability to translate non-English language studies for systematic reviews in five languages and found variations in accuracy [16]. Spanish translations demonstrated the highest correct extraction rate (93% of items correctly extracted more than half the time), followed by German and Japanese (89%), French (85%), and Chinese (78%). According to the authors, caution is advised when using machine translation, as there is a trade-off between achieving comprehensive reviews and the potential for translation-related errors.

The objective of the current study was to assess the performance of three MTs, namely *DeepL*, *Google Translate*, and *CUBBITT*, in translating medical abstracts from French to English. We aimed to compare the accuracy of translations using nine metrics of *Recall-Oriented Understudy for Gisting Evaluation* (*ROUGE*), while also considering the stylistic quality through human evaluation. This study addressed the challenges faced by non-English speaking medical researchers and explored the practicality of using machine translation in this context. By testing our hypothesis that MTs may exhibit variations in translating medical research, we aimed to provide valuable insights for French-speaking researchers seeking to publish in English-language journals.

## Methods

### Selection of abstracts and machine translators (MTs)

We selected the two most prestigious general medical journals (according to the 2020 *Journal Citation Reports* impact factor) that translate all (*Canadian Family Physician*, impact factor = 3.3) or some (*CMAJ*, impact factor = 8.3) of the abstracts of published articles into French. We limited this preliminary study to general medical journals and did not include medical specialty or basic science journals that may use more technical language. We selected high-impact journals in a bilingual English/French country (Canada) to ensure that the French abstracts included in the study were of high quality.

We randomly extracted ten articles published in 2021 with abstracts available in French, five published in *CMAJ* (abstracts #1 to #5) and five in *Canadian Family Physician* (abstracts #6 to #10). We included ten articles in the study to obtain a variety of topics and study designs. Taken together, these ten abstracts contained 12,153 words in total.

Then, in spring 2022, we selected all MTs allowing the translation of at least 5,000 characters from French to English for free. Three MTs met these criteria (i.e., *DeepL* [https://www.deepl.com/translator], *Google Translate* [https://translate.google.com], and *CUBBITT* (Charles University Block-Backtranslation-Improved Transformer Translation) [https://lindat.mff.cuni.cz/services/translation]. At the time of the study, *DeepL* was free up to 5,000 characters, and 26 languages were available for translation; *Google Translate* was also free up to 5,000 characters, and over 100 languages were supported; *CUBBITT* had no character limit, but only six languages were available, including French and English.

### Selection of metrics to evaluate the accuracy of the translation

We selected nine metrics of *Recall-Oriented Understudy for Gisting Evaluation* (*ROUGE*) [19–21], namely *ROUGE-1 recall/precision/F1-score*, *ROUGE-2 recall/precision/F1-score*, and *ROUGE-L recall/precision/F1-score*.

*ROUGE-N* measures the number of identical n-grams between the text generated by a translator and a reference text considered as the gold standard. An n-gram is a grouping of words. For example, a unigram (1-gram) consists of a single word and a bigram (2-gram) consists of two consecutive words. The reference is a human-made optimal result. Thus, for *ROUGE-1* and *ROUGE-2*, we measure the match rate of unigrams and bigrams, respectively, between the translated text and the reference. *ROUGE-L* measures the longest sequence of words that appear in the same order in both the translated text and the reference. The idea behind this metric is that a longer shared sequence indicates greater similarity between the two versions.

*ROUGE-N* and *ROUGE-L* are evaluated using three different metrics. The recall metric counts the number of identical n-grams, respectively the longest sequence of words that appear in both the translated text and the reference, divided by the total number of n-grams in the reference. It is used to verify that the translated text captures all the information contained in the reference. The precision metric is calculated in almost the same way, but, instead of dividing by the number of n-grams in the reference, it is divided by the number of n-grams in the translated text. It is used to check that the translator does not produce irrelevant words. Finally, the F1-score combines the recall and precision metrics to obtain an overall measure of translation accuracy.

Table 1 shows how to calculate the nine metrics using an example for the translated text and an example for the reference text. Implementing these metrics is easy in *Python* [20]. There are no recognized criteria defining above which scores of *ROUGE* a MT can be considered accurate. These measures are mainly used to compare MTs with each other, knowing that the higher the scores, the higher the accuracy of the translation. The main drawback of *ROUGE* is that it measures syntactic and not semantic matches. Thus, if two sequences have the same meaning, but use different words to express that meaning, the scores could be relatively low. For this reason, we also included a human evaluation of the translation performance, by analyzing the *fluency* score. This score is used to assess whether the text contains errors that native speakers would not have made or, more simply, whether the text is written in good English [22]. The best way to evaluate *fluency* is to use a multi-point *fluency* scale, with anchor text for each value [22]:

How do you judge the *fluency* of the translation?

5 → Flawless English
4 → Good English
3 → Non-Native English
2 → Disfluent English
1 → Incomprehensible

*ROUGE* and fluency were used in a large number of studies to evaluate texts. Koto et al. identified 106 studies using *ROUGE* and 45 studies examining fluency [23].

**Table 1:** Calculation of the different metrics of *Recall-Oriented Understudy for Gisting Evaluation* (*ROUGE*) using an example for the translated text and an example for the reference text.

Metric	Number of n-grams^1^ found in the translated text (“He eats drinks coffee muffins likes chocolate”)	Number of n-grams found in the reference text (“He likes chocolate”)	Number of n-grams (for *ROUGE-1* and *ROUGE-2*), or longest common subsequence (for *ROUGE-L*), found in the translated text and the reference text	*Recall* ^2^	*Precision* ^3^	*F1-score* ^4^
*ROUGE-1* ^5^	7	3	3 (i.e., “He”, “likes”, “chocolate”)	3/3 = 1	3/7 = 0.43	2*(0.43*1)/(0.43+1) = 0.6
*ROUGE-2* ^6^	6	2	1 (i.e., “likes chocolate”)	1/2 = 0.5	1/6 = 0.17	2*(0.17*0.5)/(0.17+0.5) = 0.3
*ROUGE-L* ^7^	7	3	3 (i.e., “He likes chocolate”)	3/3 = 1	3/7 = 0.43	2*(0.43*1)/(0.43+1) = 0.6

^1^ An n-gram is a grouping of words. For example, a unigram (1-gram) consists of a single word and a bigram (2-gram) consists of two consecutive words

^2^ *Recall* = number of n-grams found in the translated text and the reference divided by number of n-grams in the reference

^3^ *Precision* = number of n-grams found in the translated text and the reference divided by number of n-grams in the translated text

^4^ *F1-score* = 2 * (*precision* * *recall*) / (*precision* + *recall*)

^5^ *ROUGE-1* measures the number of matching 1-grams between the translated text and the reference

^6^ *ROUGE-2* measures the number of matching 2-grams between the translated text and the reference

^7^ *ROUGE-L* measures the longest sequence of words that appear in the same order in both the translated text and the reference. For *ROUGE-L*, we use the number of 1-grams for the denominator (number of 1-grams in the reference for recall and number of 1-grams in the translated text for precision)

### Data collection

We translated the ten abstracts from French to English using the three selected tools. The ten original abstracts in English and the versions obtained after translation by the three MTs are available elsewhere (https://doi.org/10.17605/OSF.IO/RCB36). Then, we evaluated the accuracy of the translation using the nine metrics of *ROUGE*, taking the original English abstract as reference text.

We also asked ten native English-speakers (five women and five men) to rate the *fluency* of the abstracts, including the original version, using the multi-point *fluency scale*. We added up the scores of the ten raters to get the overall score, ranging from 10 to 50. All study raters were acquaintances of the investigators, with a scientific background. In detail, there were five physicians and a fifth year medical student, two non-governmental organization (NGO) workers, a data scientist, and a manager. To avoid biasing the human evaluation, the raters were told that all versions were authored by translators in training, and the order of the versions was different for each abstract.

### Statistical analyses

The nine metrics of *ROUGE* and the *fluency* score were first recorded separately for each version of each abstract. For *ROUGE*, the number of texts analyzed was 30 (i.e., the ten abstracts for each of the three MTs), whereas for the *fluency* score, this number was 40, since the original version of the ten abstracts were also analyzed. Using medians and interquartile ranges (IQRs), the results were then summarized for each of the MTs for *ROUGE* (n = 3), and for each of the MTs and the original version for the *fluency* score (n = 4). We summarized the results using medians and IQRs, because the data did not follow a normal distribution. We used Kruskal-Wallis tests to assess whether the differences in medians were statistically significant. The assumption of a similar distribution shape for all groups was met. If there were significant differences between groups, we used Dunn tests, with adjustments for multiple comparisons (Sidak), to identify the specific groups that differed from each other.

We also extracted the number of abstracts with the highest score for the nine metrics of *ROUGE* for *DeepL*, *Google Translate*, and *CUBBITT* respectively. We did the same for the *fluency* score, for *DeepL*, *Google Translate*, *CUBBITT*, and the reference text respectively.

Finally, for the human evaluation, we examined the inter-rater agreement between the ten raters for the reference text and the versions translated by *DeepL*, *Google Translate*, and *CUBBITT*. We used the ‘kappaetc’ command in Stata to calculate the quadratic weighted agreement coefficients (percent agreement and Gwet’s AC) [24, 25]. The statistical significance was set at a two-sided p-value of ≤0.05. All analyses were performed with STATA 15.1 (College Station, USA).

### Ethical considerations

Since this study did not involve the collection of personal health-related data it did not require ethical review, according to current Swiss law.

## Results

The raw data (i.e., the scores obtained for the nine metrics of *ROUGE* and the *fluency* score) are available in *Open Science Framework* (https://doi.org/10.17605/OSF.IO/RCB36).

**Fig 1:**
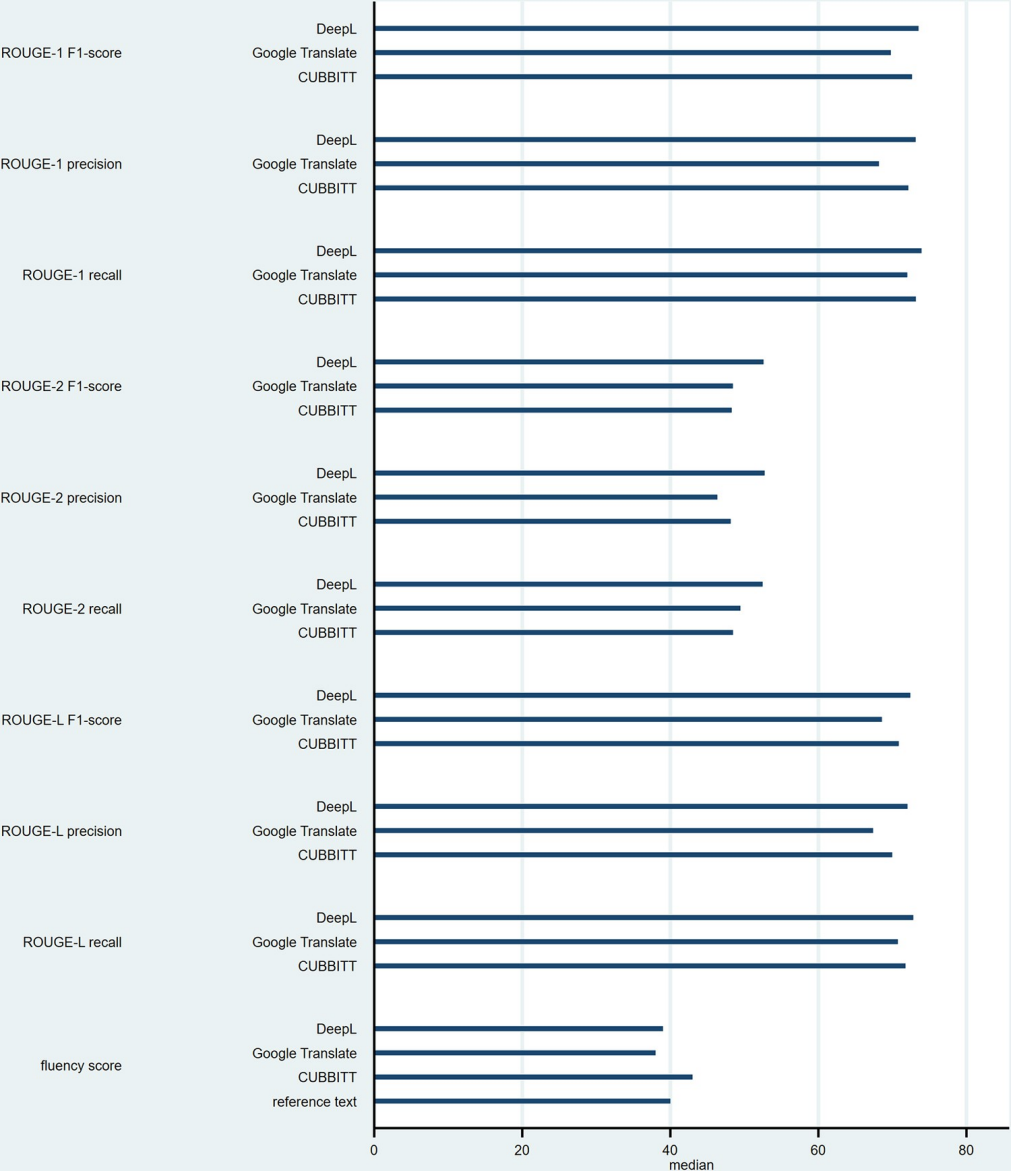
*Recall-Oriented Understudy for Gisting Evaluation (ROUGE)* and *fluency* median scores. Data are presented for the three machine translators (MTs) separately for *ROUGE*, and for the three MTs and the reference text for the *fluency* score. Median scores for *ROUGE* are presented as percentages.

Tables 2 (for *ROUGE*) and 3 (for the *fluency* score) summarize these data using medians and IQRs, as well as the number of abstracts with the highest score. Fig 1 presents the data in graphical form. *ROUGE* median scores were higher for *Deepl* than for *CUBBITT*, and higher for *CUBBITT* than for *Google Translate*, except for *ROUGE-2 F1* and *ROUGE-2 recall* for which scores were higher for *Google Translate* than for *CUBBITT*. However, none of these differences was statistically significant (medians ranging from 0.5246 to 0.7392 for *DeepL*, from 0.4634 to 0.7200 for *Google Translate*, and from 0.4815 to 0.7316 for *CUBBITT*, all p-values > 0.10). For the overall *fluency* score (we added up the scores of the ten raters to get this score), *CUBBITT* tended to score higher than *DeepL*, *Google Translate*, and the original English text (median = 43 for *CUBBITT*, vs. 39 for *DeepL*, 38 for *Google Translate*, and 40 for the original English text, p-value = 0.003). The difference in median score was borderline significant between *CUBBITT* and the reference text (p-value = 0.05), whereas it was statistically significant between *CUBBITT* and *DeepL* (p-value = 0.03), and between *CUBBITT* and *Google Translate* (p-value = 0.001). All ten abstracts received an individual score ranging from 3 to 5 (no score was below 3), even *Google Translate*, which achieved the lowest overall median score.

**Table 2:** Median score (IQR) and number of abstracts with the highest score for the nine *Recall-Oriented Understudy for Gisting Evaluation* (*ROUGE*) metrics used to evaluate translations of ten abstracts of medical scientific articles by three machine translators (*DeepL*, *Google Translate*, and *CUBBITT*).

Machine translator (MT)	*ROUGE-1 F1-score*	*ROUGE-1 precision*	*ROUGE-1 recall*	*ROUGE-2 F1-score*	*ROUGE-2 precision*	*ROUGE-2 recall*	*ROUGE-L F1-score*	*ROUGE-L precision*	*ROUGE-L recall*
*DeepL*									
Median score (IQR) for the ten abstracts	0,7352 (0.1298)	0,7312 (0,1077)	0,7392 (0,1524)	0,5259 (0,1700)	0,5273 (0.1743)	0,5246 (0.1677)	0,7241 (0.1370)	0,7202 (0.1122)	0,7281 (0.1625)
Number (%) of abstracts with the highest score	6 (60.0)	7 (70.0)	7 (70.0)	8 (80.0)	9 (90.0)	7 (70.0)	6 (60.0)	8 (80.0)	6 (60.0)
*Google Translate*									
Median score (IQR) for the ten abstracts	0,6978 (0.0740)	0,6817 (0.0549)	0,7200 (0.0881)	0,4845 (0.0804)	0,4634 (0.0921)	0,4947 (0.0852)	0,6856 (0.0933)	0,6738 (0.0779)	0,7073 (0.1047)
Number (%) of abstracts with the highest score	1 (10.0)	0	2 (20.0)	0	0	1 (10.0)	2 (20.0)	0	2 (20.0)
*CUBBITT*									
Median score (IQR) for the ten abstracts	0,7264 (0.1273)	0,7214 (0.0990)	0,7316 (0.1349)	0,4828 (0.1456)	0,4815 (0.1425)	0,4846 (0.1488)	0,7084 (0.1433)	0,6995 (0.1103)	0,7175 (0.1513)
Number (%) of abstracts with the highest score	3 (30.0)	3 (30.0)	1 (10.0)	2 (20.0)	1 (10.0)	2 (20.0)	2 (20.0)	2 (20.0)	2 (20.0)
p-value ^1^	0.59	0.18	0.70	0.43	0.27	0.68	0.62	0.30	0.76

^1^ Kruskal-Wallis tests to assess whether differences in median scores between the three MTs were statistically significant

**Table 3:** Median *fluency score* (IQR) and number of abstracts with the highest score. This score was used to assess the style of ten original English abstracts (= reference text) and the versions translated by *DeepL*, *Google Translate*, and *CUBBITT*.

Reference text or machine translator (MT)	*Fluency score* ^1^^,^^2^ Median (IQR) or N (%)
Reference text	
Median score (IQR) for the ten abstracts	40 (4)
Number (%) of abstracts with the highest score	0
*DeepL*	
Median score (IQR) for the ten abstracts	39 (3)
Number (%) of abstracts with the highest score	2 (20)
*Google Translate*	
Median score (IQR) for the ten abstracts	38 (1)
Number (%) of abstracts with the highest score	0
*CUBBITT*	
Median score (IQR) for the ten abstracts	43 (4)
Number (%) of abstracts with the highest score	8 (80)

^1^ The evaluation was done independently by ten native English-speaking raters. They were asked to answer to the question “How do you judge the fluency of the translation?” Possible answers were 5 (= flawless English), 4 (= good English), 3 (= non-native English), 2 (= disfluent English), 1 (= incomprehensible). We added up the scores of the ten raters to get the overall score, ranging from 10 to 50.

^2^ Kruskal-Wallis or Dunn tests to assess whether differences in median scores between the groups were statistically significant. P-value for the difference between the four groups = 0.003, between *CUBBITT* and the reference text = 0.05, between *CUBBITT* and *DeepL* = 0.03, between *CUBBITT* and *Google Translate* = 0.001, between the reference text and *DeepL* = 0.95, between the reference text and *Google Translate* = 0.45, between *DeepL* and *Google Translate* = 0.62.

*ROUGE* scores were highest for six to nine abstracts for *DeepL* (depending on the score considered), one to three abstracts for *CUBBITT*, and zero to two abstracts for *Google Translate*. *Fluency* scores were highest for eight abstracts for *CUBBITT*, two abstracts for *DeepL*, and zero abstracts for *Google Translate* and the reference text.

Finally, the inter-rater agreement between the ten raters was high (Table 4). The raters agreed on more than 97% of the abstracts (p-values < 0.001), and the chance-corrected Gwet’s agreement coefficients were high (p-values < 0.001).

**Table 4:** Inter-rater agreement between the ten native English-speaking raters who evaluated the style of ten original English abstracts (= reference text) and the versions translated by *DeepL*, *Google Translate*, and *CUBBITT*.

Reference text or machine translator (MT)	Percent agreement (95%CI)	p-value	Gwet’s AC (95%CI)	p-value
Reference text	0.9789 (0.9691–0.9886)	<0.001	0.9667 (0.9437–0.9897)	<0.001
*DeepL*	0.9792 (0.9696–0.9887)	<0.001	0.9684 (0.9476–0.9892)	<0.001
*Google Translate*	0.9786 (0.9699–0.9873)	<0.001	0.9665 (0.9466–0.9865)	<0.001
*CUBBITT*	0.9739 (0.9679–0.9799)	<0.001	0.9530 (0.9364–0.9696)	<0.001

The ten raters were asked to answer to the question “How do you judge the fluency of the translation?” Possible answers were 5 (= flawless English), 4 (= good English), 3 (= non-native English), 2 (= disfluent English), 1 (= incomprehensible). We calculated the quadratic weighted agreement coefficients (percent agreement and Gwet’s AC)

## Discussion

### Main findings

We compared the performance of three MTs (i.e., *DeepL*, *Google Translate*, and *CUBBITT*) for translating medical research abstracts from French to English. In this preliminary study, we evaluated five abstracts published in *CMAJ* and five abstracts published in *Canadian Family Physician*. We found that the three MTs performed similarly when tested with *ROUGE*, but *CUBBITT* was slightly better than the other two using human evaluation. We also found that for human evaluation *CUBBITT* tended to perform better than the original English text.

### Comparison with existing literature

MTs are increasingly used in medicine, particularly to translate electronic medical records and improve patient care [3, 7–15]. Current evidence also suggests that they are relatively reliable for extracting data from non-English articles in systematic reviews [16, 17]. However, there is little data available on their effectiveness in academic research. Using a subjective evaluation method, Takakusagi et al. investigated the accuracy of DeepL in translating an entire medical article from Japanese into English [26]. The authors compared the original Japanese article with the English version, which was back translated into Japanese by medical translators. They found that the overall accuracy was high, with an average match rate of 94%. However, the accuracy varied between sections of the article, with the ’Results’ section showing the highest accuracy (100%) and the ’Materials and Methods’ section showing the lowest accuracy (89%). The authors limited their analysis to the accuracy of the meanings and did not assess the stylistic quality of the translation.

In our study, we found that *DeepL*, *Google Translate*, and *CUBBITT* are three effective tools for accurately and fluently translating abstracts of medical articles from French into English. Surprisingly, *CUBBITT* did better in terms of *fluency* as the original English abstracts published in two high-impact English-language journals. These results are however in line with a recent study conducted by the developers of *CUBBITT*, which showed that the quality of translations done with this tool approached that of professional translators in terms of *fluency* [27].

Unlike the biomedical sciences, a large amount of data on machine translation is available in the field of educational linguistics, second language studies, and foreign language education. Two review articles have recently been published [28, 29]. These papers summarized the key concepts, insights, and findings, categorizing them into questions like how learners use MTs, what instructors and learners think about MTs, and how MTs affect language learning. Students have diverse opinions concerning the appropriateness, reliability, and ethical considerations of machine translation tools [30–33]. Learners generally hold favorable views of machine translation, believing it has the potential to assist their learning and enhance the quality of their second language writing. However, these positive perceptions are counterbalanced by concerns about machine translation accuracy, an understanding of its limitations, and conflicting interpretations of what constitutes ethical behavior. The literature exploring the potential advantages of machine translation in language learning did not produce definitive findings. However, it suggests two potential trends: MTs might serve as a valuable resource for improving learners’ metalinguistic understanding [34–37], and they can aid students in achieving better results in translation and second language writing tasks [38, 39]. Some of these studies focused on the use of *Google Translate* [40–42], yet neither review included studies that made direct comparisons between MTs.

To our knowledge, few comparative studies are available in the literature. Hidalgo-Ternero assessed the performance of *Google Translate* and *DeepL* in translating Spanish idiomatic expressions into English, including both common and less frequent variants, with a focus on whether these idioms were presented in continuous or discontinuous forms [43]. The study found that *Google Translate* and *DeepL* performed well in accurately translating high-frequency idiomatic expressions, achieving an average accuracy rate of 86% and 89%, respectively. However, they struggled to detect and translate lower-frequency phraseological variants of these idioms, indicating limitations in handling less common idiomatic expressions. Focusing on human post-editing efforts, another study compared the performance of three MTs for translating Cochrane plain language health information from English to Russian [13]. The authors found that Google Translate performed best, slightly better than DeepL, while Microsoft Translator performed less well.

Our study was not designed to estimate the amount of time needed by researchers for post-editing (i.e., the time needed to make corrections to the text after it has been translated into English by the MT). Given the results obtained for the *fluency* score, post-editing should nevertheless be performed fairly quickly. Indeed, even without post-editing, the stylistic quality of the translation was considered by the evaluators to be better (for *CUBBITT*) or almost as good (for *DeepL* and *Google Translate*) as the original text.

This preliminary study included only abstracts, which are generally written to be more accessible and more quickly “digested” than full articles. We did not evaluate the performance of MTs with full articles. Translation apps often lack specialized medical terminology, which can make them useless for translating highly specialized medical articles. Further studies evaluating the performance of MTs for full articles and for various disciplines are therefore needed. However, we believe that non-English-speaking researchers who do not wish to rely on the services of professional translators (e.g. because of their cost) could have an interest in using *DeepL*, *Google Translate*, or *CUBBITT* for some of their work that is not highly specialized. Indeed, the time spent in post-editing after using these MTs probably be far outweighed by the time they would have to spend translating scientific articles themselves or the time spent writing the articles directly in English.

### Strengths and limitations

Our study has several strengths. We incorporated a dual assessment approach, combining quantitative *ROUGE* metrics and qualitative *fluency* evaluations by native English speakers. This ensures a comprehensive evaluation of machine translation tools, providing a nuanced understanding of both syntactic and semantic aspects of the translations. In addition, focusing on medical texts, our method aligns with practical scenarios faced by non-English-speaking researchers. By evaluating tools in a domain-specific context, our approach offers insights directly applicable to researchers in the medical field, enhancing the relevance of the study. Finally, the inclusion of raters with varied scientific backgrounds enhances the robustness of the fluency assessment. This diversity ensures a broad perspective on the quality of translations, considering the expectations and language nuances across different professional domains.

However, our study also has some weaknesses. First, we included only French abstracts published in two general medical journals. It is not certain that the results would have been similar for full articles, other languages, and/or other journals. The selection of two bilingual journals introduces a potential limitation as the study’s outcomes rely on the quality of translated abstracts from English to French published in these journals. While the stylistic quality of these versions was generally deemed good or excellent by the raters, it is essential to acknowledge the influence of the initial French abstracts on the translation process. Second, although *ROUGE* is a validated instrument that is often used to evaluate the performance of MTs, it does not measure semantic matches. If two sequences have the same meaning, but use different words to express that meaning, the score assigned could be relatively low. Third, only ten abstracts were included in the study and only ten raters were recruited for the human evaluation. We included only ten abstracts, because it was important for the evaluators to carefully assess the four versions of the abstracts (the original version and the versions from *DeepL*, *Google Translate*, and *CUBBITT*), and this was a time-consuming task. Future studies may consider including a larger sample to obtain more robust results. Finally, we selected abstracts from the year 2021 to ensure that the texts were current and reflected the latest developments in medicine. Future studies may encompass a broader time frame to examine variations over the years.

## Conclusion

Our study provides a thorough examination of the performance of MTs—*DeepL*, *Google Translate*, and *CUBBITT*—in the specific context of translating medical research abstracts from French to English. This focused evaluation contributes to a nuanced understanding of the applicability of these tools in the medical domain. We not only assessed the accuracy of translations using established metrics but also delved into the fluency of the translated text. Our study aims to highlight the practical utility of MTs for non-English-speaking researchers in medicine.

We found that the three MTs performed similarly when tested with *ROUGE*, but *CUBBITT* was slightly better than the other two using human evaluation. We also found that in terms of stylistic quality *CUBBITT* tended to perform better than the original English text.

Although the study was limited to the analysis of abstracts published in general medical journals and did not evaluate the time required for post-editing, we believe that French-speaking researchers could benefit from using *DeepL*, *Google Translate*, or *CUBBITT* to translate articles written in French into English. Further studies would be needed to evaluate the performance of MTs with full articles and languages other than French.
